# Paucimannosidic glycoepitopes inhibit tumorigenic processes in glioblastoma multiforme

**DOI:** 10.18632/oncotarget.27056

**Published:** 2019-07-09

**Authors:** Yvonne Becker, Sarah Förster, Gerrit H. Gielen, Ian Loke, Morten Thaysen-Andersen, Christine Laurini, Kristin Wehrand, Torsten Pietsch, Simone Diestel

**Affiliations:** ^1^ Institute of Nutrition and Food Sciences, Department of Human Metabolomics, University of Bonn, Bonn 53115, Germany; ^2^ Institute of Neuropathology, University of Bonn Medical Center, Bonn 53127, Germany; ^3^ Department of Molecular Sciences, Macquarie University, Sydney, NSW 2109, Australia

**Keywords:** cancer progression, glioblastoma multiforme, N-glycosylation, paucimannosidic epitopes, AHNAK

## Abstract

Glioblastoma multiforme is an aggressive cancer type with poor patient outcomes. Interestingly, we reported previously a novel association between the little studied paucimannosidic *N*-linked glycoepitope and glioblastoma. Paucimannose has only recently been detected in vertebrates where it exhibits a very restricted tumor-specific expression. Herein, we demonstrate for the first time a very high protein paucimannosylation in human grade IV glioblastoma and U-87MG and U-138MG glioblastoma cells. Furthermore, we revealed the involvement of paucimannosidic epitopes in tumorigenic processes including cell proliferation, migration, invasion and adhesion. Finally, we identified AHNAK which is discussed as a tumor suppressor as the first paucimannose-carrying protein in glioblastoma and show the involvement of AHNAK in the observed paucimannose-dependent effects. This study is the first to provide evidence of a protective role of paucimannosylation in glioblastoma, a relationship that with further *in vivo* support may have far reaching benefits for patients suffering from this often fatal disease.

## INTRODUCTION

Glioblastoma multiforme (GBM) is the most aggressive type of glioma. Gliomas arise from glia or precursor cells in the central nervous system and are clinically classified into four grades (I–IV). GBM is considered as a grade IV tumor and the most common malignant primary brain tumor in adults. A median survival of only 14 to 16 months after diagnosis has been reported. However, 3–5% of GBM patients survive for more than 3 years due to better responsiveness to available therapeutic compounds, calling for customized treatment strategies for GBM patients [1–5]. Development of efficacious therapies against GBM is challenging due to the cellular heterogeneity of the tumor tissue. Research is intensifying to identify reliable biomarkers to enable earlier diagnosis of GBM in order to increase patient survival and to find cure for the disease. In recent years several potential diagnostic, prognostic and predictive biomarkers have been proposed [6]. So far, proteins involved in DNA methylation, oncogenes like epidermal growth factor and vascular endothelial growth factor and their respective receptors as well as tumor suppressor proteins like p53 and Phosphatase and Tensin homolog PTEN, and different phospholipid metabolites have been discussed as potential biomarkers for GBM [7].

Glycosylation is one of the most frequent co- and posttranslational modifications with approximately 50% of all proteins being glycosylated. It is a critical determinant for correct protein function with important roles in various cellular processes including cell proliferation, migration and adhesion. Defects in the glycosylation machinery often results in detrimental and even fatal cellular consequences highlighting the biological significance of protein glycosylation [8]. Glycans are commonly attached to asparagine residues (*N*-linked glycosylation) of proteins [9]. Due to the involvement of glycoproteins in various processes, it is not surprising that changes in protein glycosylation are associated with cancer development, progression and malignancy by regulating tumor proliferation, survival, invasion, metastasis and angiogenesis [10–12]. For example, changes in the terminal glycan features e. g. sialylation and fucosylation and aberrations in the underpinning glycosylation machinery have frequently been described [8, 13].

The human glycobiological literature harbors an impressive body of knowledge of the three conventional *N*-glycosylation types (i. e., oligomannosidic, hybrid and complex glycans) in vertebrates [8]. A few years ago a much less reported type of protein *N*-glycosylation, known as paucimannosylation was discovered in vertebrates. Paucimannose consists of two *N*-acetylglucosamine, 1–3 mannose residues and the variable presence of a fucose residue (mannose_1–3_fucose_0–1_ *N*-acetylglucosamine_2_ [14]). Interestingly, its presence seems to be restricted to some pathophysiological conditions e. g. cancer or pathogen infected macrophages [15–19] and to specific stem cell populations like early postnatal neural stem cells [20, 21]. These interesting observations and the fact that paucimannose is seemingly negligible or absent under normal basal conditions of human physiology [22] make protein paucimannosylation a highly interesting candidate for cancer diagnosis and for novel therapeutic purposes.

Previously, we reported that paucimannosidic epitopes influence cell proliferation of the glioblastoma cell line A172 [21], providing a first evidence that protein paucimannosylation might influence tumorigenic processes. Herein, we aimed to further investigate the functional involvement of paucimannose in a variety of tumorigenic processes in GBM in two glioblastoma cell lines, U-87 MG and U-138 MG, and in human patient tissue. We report here that protein paucimannosylation is present at high level in human GBM tissue compared to healthy tissue, and plays a central role in GBM progression by inhibiting cell proliferation, migration and invasion of cancerous glial cells, most likely via the neuroblast differentiation associated protein AHNAK. AHNAK (meaning “giant” in Hebrew) or desmoyokin consists of 5890 amino acids and has a molecular weight of 629 kDa. Interestingly, its involvement in different tumorigenic processes has already been described [23].

Altogether, the results presented here propose protein paucimannosylation as an attractive biomarker for and may have value as a potential therapeutic agent against GBM.

## RESULTS

### Paucimannose levels correlate with the proliferation of glioblastoma cell lines

To investigate the levels of paucimannose in correlation to different cancerogenic properties two glioblastoma cell lines were chosen displaying differences in their tumorigenicity and proliferation rate. U-87 MG cells are highly proliferating cells with 29.8 hours doubling time [24] and are tumorigenic in immunosuppressed mice (American Type Culture Collection). In contrast, U-138 MG cells proliferate slowly (70 hours doubling time) and are non-tumorigenic in immunosuppressed mice (Deutsche Sammlung für Mikroorganismen und Zellkulturen). Therefore, tumors of different aggressiveness are represented in these two selected cell lines. Since proliferation rates may vary due to different culture conditions the doubling time of U-87 MG and U-138 MG cells under our chosen conditions was determined first. The doubling time of U-87 MG cells with 26.5 hours was comparable to the previously described one, whereas U-138 MG cells showed a faster doubling time of 48.4 hours than earlier described (data not shown). However, the doubling times of both cell lines were still different from each other, being almost twice as fast for U-87 MG cells compared to U-138 MG cells.

**Figure 1:**
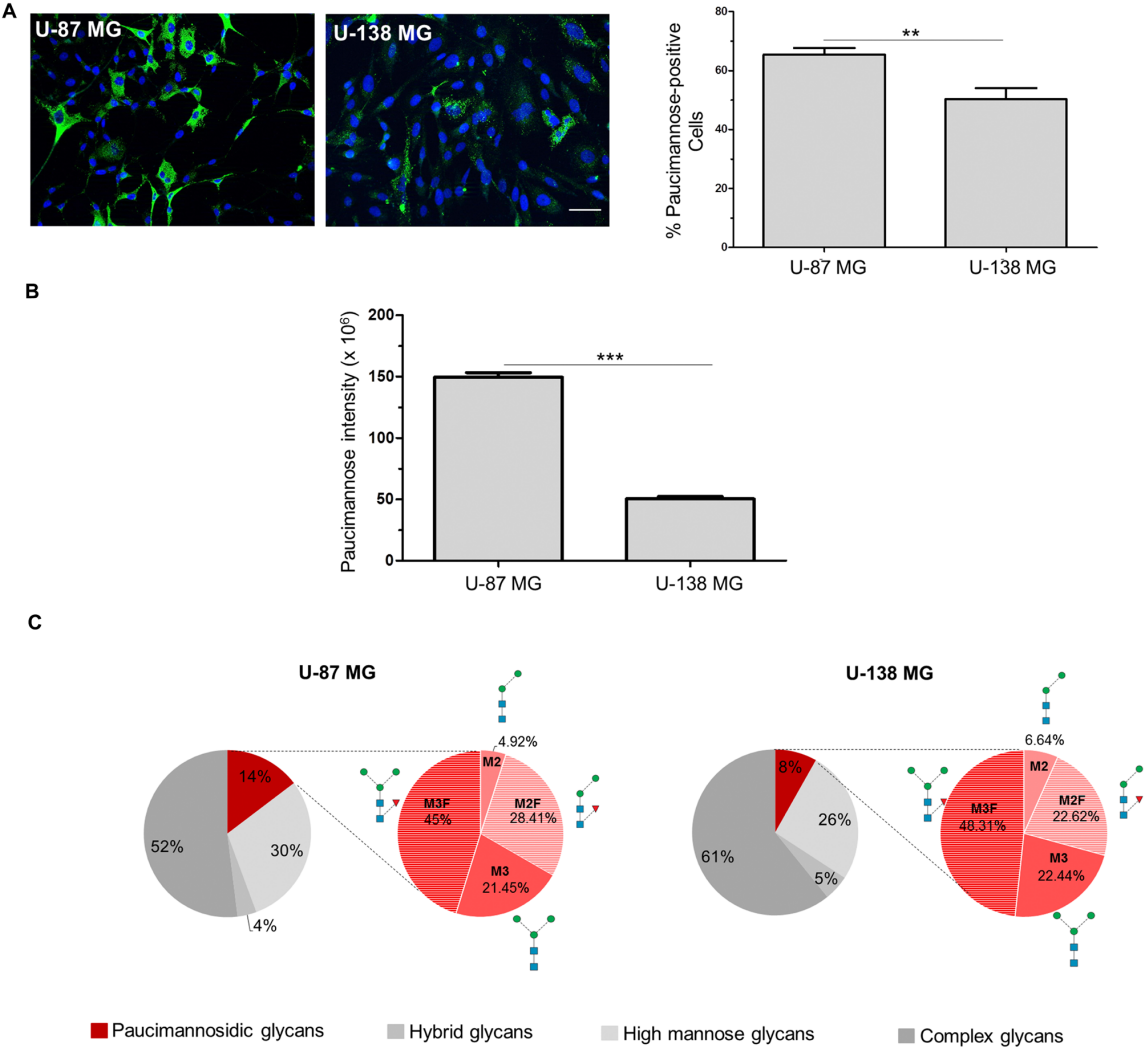
Paucimannosidic epitopes differ in U-87 MG and U-138 MG cells. (**A, B**) U-87 MG or U-138 MG cells were stained with Mannitou antibody and Cy2-conjugated secondary antibodies (green). Cells were counterstained with Hoechst 33258. (**A**) Representative images of U-87 MG or U-138 MG cells, respectively, bar = 50 μm (left panel), and quantification of paucimannose-positive cells are shown (right panel), data are given as mean ± SEM. (**B**) Quantification of paucimannose-signal intensity of paucimannose-positive cells. At least 15 images were taken from three independent experiments, data are given as mean ± SEM. (**C**) *N*-Glycome profiling of the protein extracts of total cell lysates of U-87 MG and U-138 MG cells. The distribution (in %) of the four major *N*-glycan types of the total *N*-glycome is indicated. The relative abundance of paucimannosidic *N*-glycans is shown in red, the abundance of the different paucimannosidic structures is shown in the red pie chart.

Next, the relative paucimannose levels in these cell lines were compared using a paucimannose-specific antibody (hereafter “Mannitou”). Interestingly, 65.3% ± 2.3% U-87 MG cells were paucimannose-positive compared to 50.3% ± 3.8% of the U-138 MG cells (Figure 1A). Furthermore, the paucimannose signal intensity of positive cells was investigated. Here, a significantly higher signal intensity of 149.5 × 10^6^ ± 3.6 × 10^6^ pixel could be observed in U-87 MG cells compared to U-138 MG cells with 50.4 × 10^6^ ± 1.8 × 10^6^ pixel (Figure 1B). To confirm the differential expression levels in the two cell lines, *N*-glycomics analysis was performed to show that paucimannosidic glycans constitute 14.66% ± 2.46% of the total *N*-glycome in U-87 MG cell lysates, whereas the cell lysates of U-138 MG cells only comprised 8.17% ± 3.82% paucimannosidic glycans of the total *N*-glycome. The ratio of single paucimannosidic structures does not differ significantly between U-87 MG and U-138 MG cells (Figure 1C). Paucimannose levels of the microsomal fractions (mostly encompassing intracellular membrane glycoproteins from intracellular organelles, mainly ER) were similar across the two cell lines (Supplementary Figure 1A), suggesting that differential paucimannosylation may be restricted to soluble luminal and plasma membrane glycoproteins. *N*-glycan analysis revealed also no difference in sialylation between U-87 MG and U-138 MG cell lysates although sialylation changes have been implicated in tumorigenic processes [8]. The fucosylation of *N*-glycans seems reduced in U-138 MG cells compared to U-87 MG cells consistent with data showing that enhanced fucosylation promotes tumor progression in lung cancer [25] (Supplementary Figure 1B). Interestingly, complex glycans were less present in cell lysates of U-87 MG cells (51.96% ± 15.33% of the total *N*-glycome), compared to cell lysates of U-138 MG cells with 60.83% ± 13.43% complex glycans (Figure 1C). No obvious difference in the levels of high mannose and hybrid glycans could be observed between the cell lysates of U-87 MG and U-138 MG (Figure 1C).

**Figure 2:**
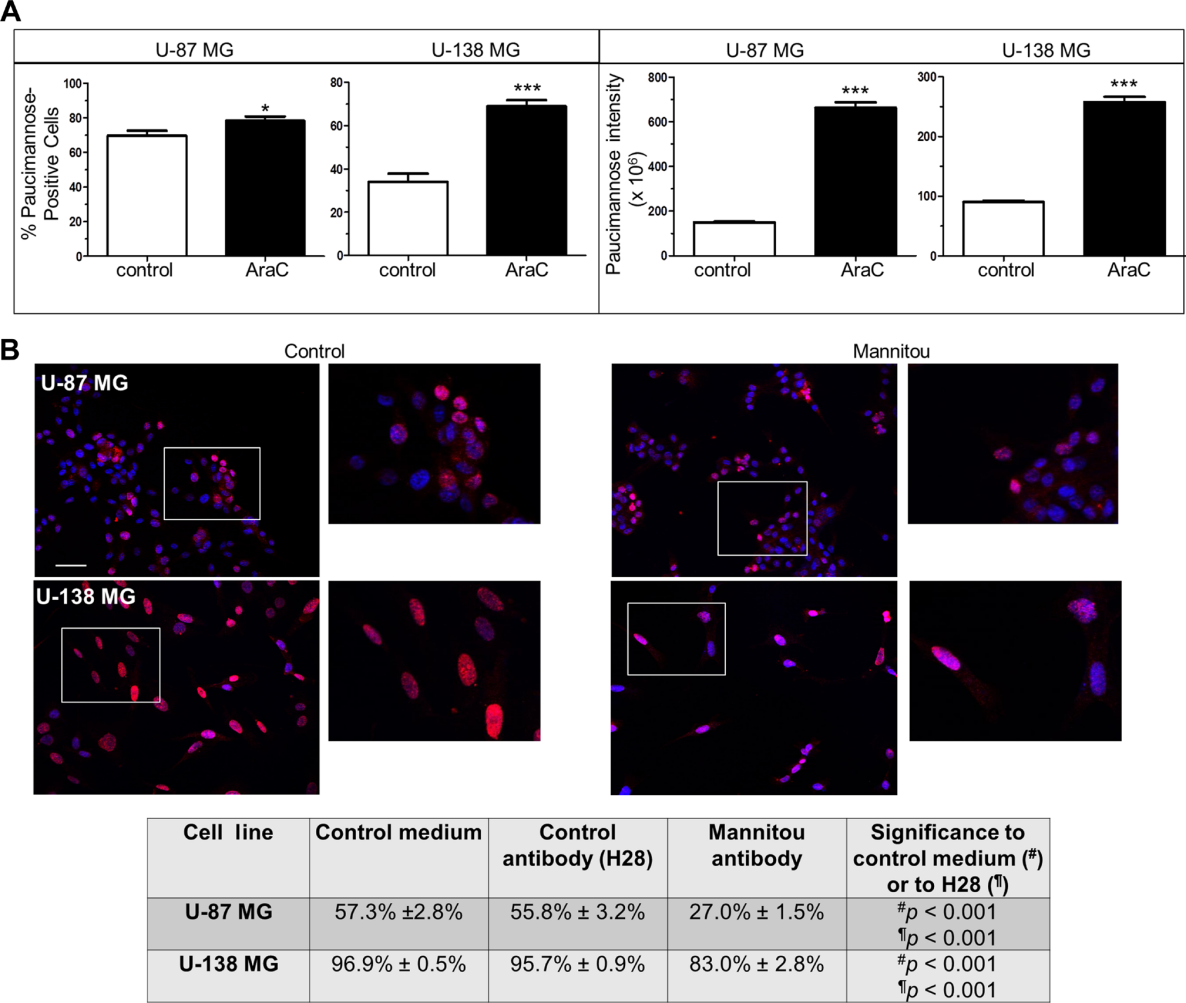
Paucimannosidic epitopes are involved in proliferation of U-87 MG and U-138 MG cells. (**A**) U-87-MG or U-138 MG cells were cultured in the presence or absence of AraC (10 μM, three days). Left panel: the number of paucimannose-positive cells was counted and referred to the total cell number. Right panel: all paucimannose-positive cells were analyzed for fluorescence intensity using Image J software. At least 15 images from three independent experiments were analyzed, data are given as mean ± SEM. (**B**) Cells were cultured for three days in control medium, control antibody hybridoma supernatant (H28) or Mannitou hybridoma supernatant, respectively. Cells were then fixed, permeabilized and stained with anti-Ki67 antibodies and anti-rabbit Cy3-conjugated secondary antibodies (red). Nuclei were counterstained with Hoechst 33258. The number of Ki67-positive cells was counted and referred to the total cell number. Top: representative images of U-87 MG cells and U-138 MG cells in control medium or Mannitou supernatant, bar = 50 μm. Bottom: quantification of Ki67-positive cells in different culture conditions. At least 15 images from three independent experiments were analyzed and data are given as mean ± SEM.

Since we have previously shown a relationship between protein paucimannosylation and cell proliferation using the A172 glioblastoma cell line [21], we speculated that the different paucimannose levels observed in U-87 MG and U-138 MG cells could be due to different proliferation of cells and/or different tumorigenicity. To test this hypothesis, the proliferation was inhibited using β-D-arabinofuranoside (AraC) and paucimannose levels were again investigated. In agreement with our previous findings, we found a correlation between paucimannose levels and proliferation of cells. U-87 MG cells, which showed already a high percentage of paucimannose-positive cells under control conditions (69.8% ± 2.8%), exhibited a moderate but significant increase after AraC incubation to 78.4% ± 2.5% (Figure 2A). Importantly, the paucimannose level per cell was drastically increased to 443.5% ± 16.4% by AraC treatment compared to the control (100% ± 2.4%, Figure 2A). Similar results were obtained for U-138 MG cells, with a significant increase in paucimannose-positive cells from 34.2% ± 3.7% (control) to 69% ± 2.8% (AraC treatment) and in the paucimannose level per cell (100% ± 2.5% in the control and 284.2% ± 9.7% in the AraC treated cells) (Figure 2A).

### Mannitou antibody influences cell proliferation

To further investigate the role of paucimannose in cell proliferation and to investigate if a manipulation of paucimannosidic epitopes influences cell proliferation in tumorigenic and non-tumorigenic (in immunosuppressed mice) glioblastoma cells, U-87 MG and U-138 MG cells were cultured in the presence of Mannitou antibody. We demonstrated earlier that Mannitou antibody can modulate cell function [21]. As controls, cells were cultured in the presence of a control antibody without an epitope on human cells (H28 [21],) or in control medium, and Ki67-staining was performed. Ki67 is a protein required for cell mitosis and a widely used marker for cell proliferation. Incubation of cells in the presence of Mannitou antibody significantly reduced the number of Ki67-positive cells compared to both controls in both cell lines, supporting a role of paucimannose in cell proliferation (Figure 2B). U-87 MG cells showed a reduction to 27% ± 1.5% proliferating cells compared to the control with 57.3% ± 2.8%. Similarly, 96.9% ± 0.5% U-138 MG cells proliferated in the control condition whereas 83% ± 2.8% showed proliferation upon Mannitou antibody treatment.

### Mannitou antibody influences cell migration and invasion

**Figure 3:**
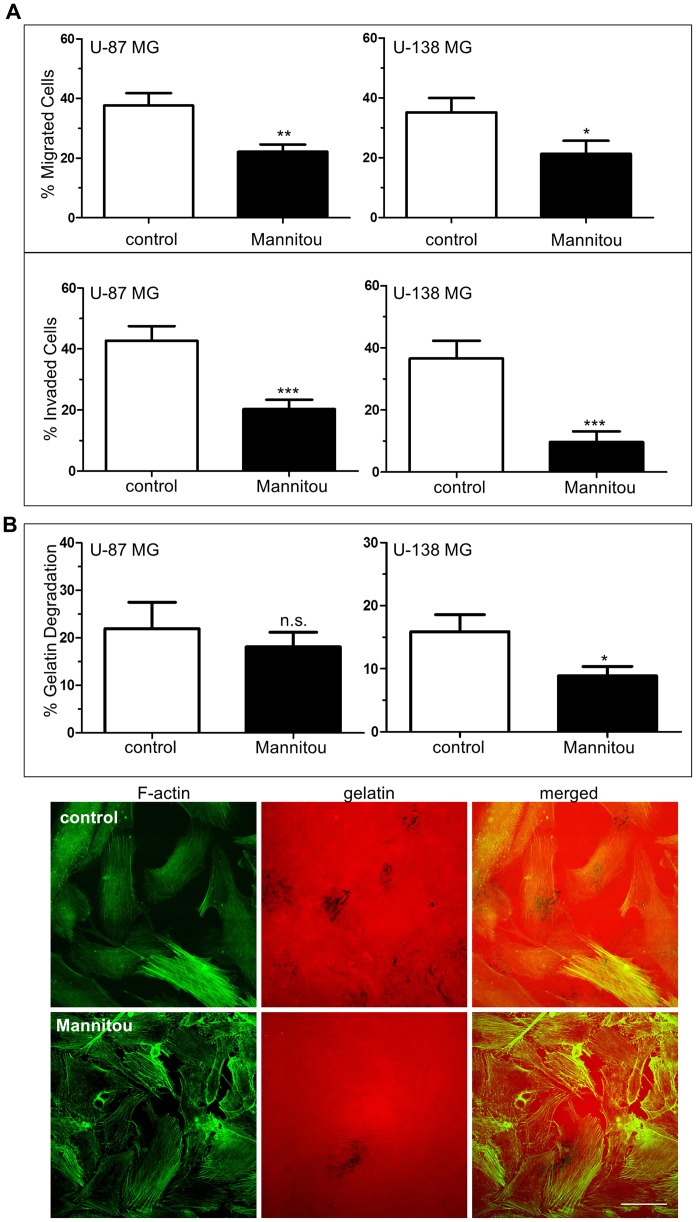
Mannitou antibody reduces haptotactic cell migration and invasion. (**A**) Cells were cultured for three days either in control medium or in Mannitou hybridoma supernatant. Haptotactic migration towards 15% FCS (top, without Matrigel) and invasion (bottom, with Matrigel) was measured for indicated time periods see (Materials and Methods) and detected by Hoechst 33258 staining. Data are given as mean ± SEM (**B**) Cells were cultured for three days in control medium or Mannitou supernatant followed by culture for 20 hours on Cy-3-labelled gelatin (red). Cells were then fixed and stained with FITC-Phalloidin (green). Degradation of gelatin was quantified. Top: quantification of gelatin degradation from three independent experiments, data are given as mean ± SEM; bottom: representative images of U-138 MG cells plated on Cy-3-labelled gelatin, black background represents gelatin degraded areas, bar = 50 μm, n.s. = not significant.

In addition to altered cell proliferation, changes in cell migration and invasion are hallmarks of tumor aggressiveness. Therefore, these properties were also investigated in the presence and absence of Mannitou antibody. Interestingly, incubation of U-87 MG or U-138 MG cells with Mannitou antibody, respectively, significantly reduced haptotactic cell migration towards FCS as well as invasion through Matrigel. The percentage of migrating U-87 MG cells was reduced to 22.1% ± 2.4% in the presence of Mannitou antibody compared to the control which showed a migration capacity of 37.6% ± 4.2%. Similarly, only 21.3% ± 4.4% of U-138 MG cells migrated in the presence of Mannitou antibody, whereas 35.2% ± 4.8% of the control treated U-138 MG cells migrated (Figure 3A, top). The same effect was observed for invasion through Matrigel which was significantly reduced after incubation of U-87 MG and U-138 MG cells in the presence of Mannitou antibody. For U-87 MG cells, a reduction from 42.7% ± 4.8% to 20.3% ± 3.1% was observed, whereas only 9.7% ± 3.4% of U-138 MG cells invaded the Matrigel in the presence of Mannitou antibody compared to 36.6% ± 5.7% in the control condition (Figure 3A, bottom).

Cell invasion includes several key processes, such as degradation of proximal extracellular matrix (ECM) and adhesion of cells [26]. We therefore investigated the role of paucimannose in a gelatin-degradation assay. For U-138 MG cells we found a significant reduction of gelatin degradation in the presence of Mannitou antibody to 8.9% ± 1.5% compared to the control with 15.9% ± 2.7% gelatin degradation, representing a reduction of almost 50%. No significant effect was observed for U-87 MG cells, however, a tendency towards a reduction of gelatin degradation was visible (Figure 3B).

### Changes in cell migration and invasion are associated with actin cytoskeletal rearrangements

**Figure 4:**
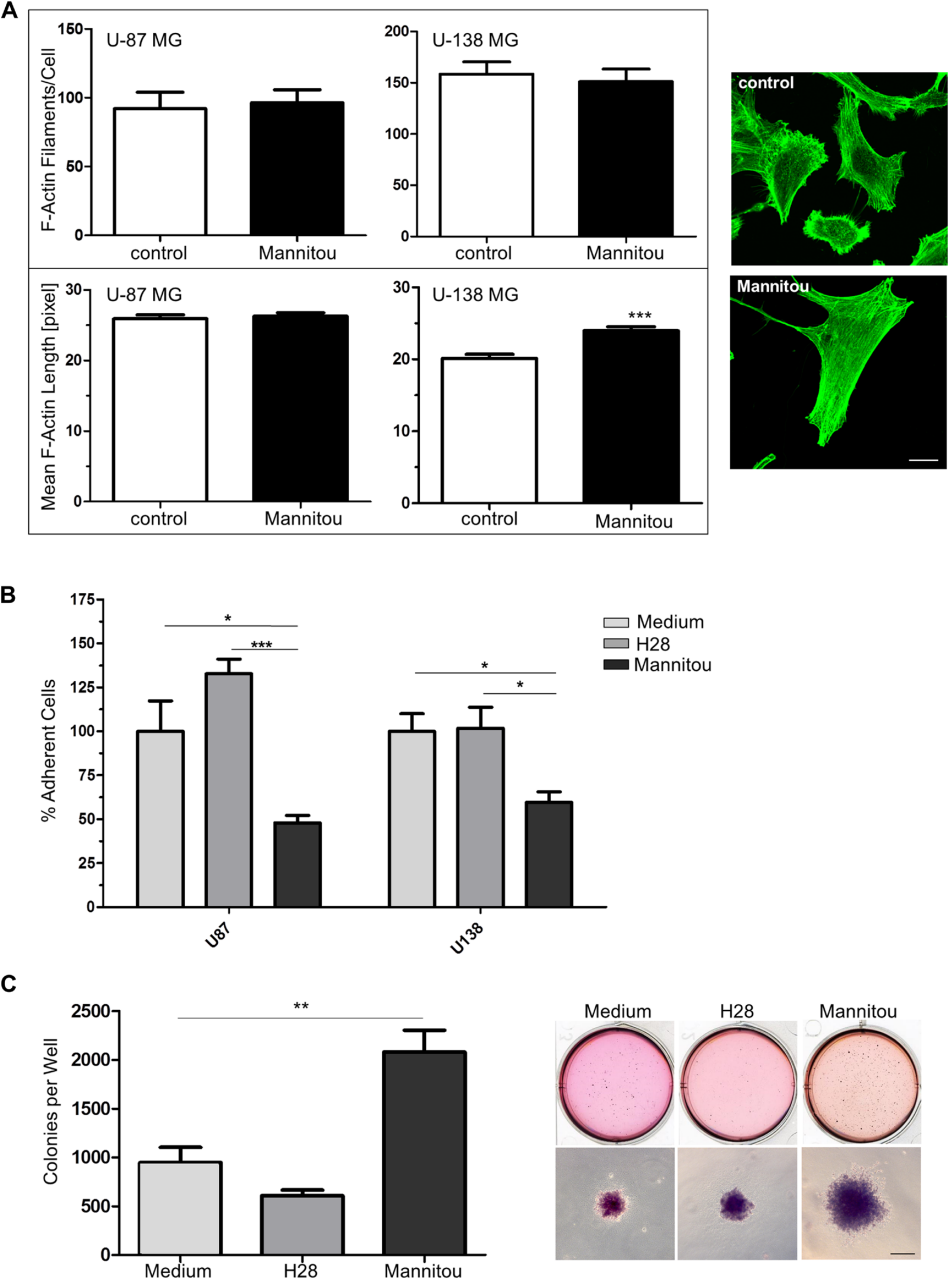
Mannitou antibody regulates actin cytoskeletal organization, cell adhesion and colony formation. (**A**) U-87 MG or U-138 MG cells were cultured for three days in control medium or Mannitou hybridoma supernatant and stained with Alexa Fluor 488 Phalloidin (green). F-actin filaments per cell and the mean F-actin length were determined using Filaquant software, left panel: quantification of F-actin filaments/cells and F-actin length, data are given as mean ± SEM; right panel: representative images of U-138 MG cells, bar = 20 μm. (**B**) Cells were cultured for three days in the presence of Mannitou antibody, H28 control antibody or in control medium and were re-plated onto 24-well plates. Adherence was determined by removing unattached cells and staining remaining cells with crystal violet followed by measurement of optical density at 550 nm. Data are collected from three independent experiments and ODs were measured in duplicate, data are given as mean ± SEM. (**C**) U-87 cells were subjected to a soft agar colony formation assay in the presence of Mannitou antibody, H28 control antibody or in control medium. The quantification of three independent experiments is shown in the left diagram, data are given as mean ± SEM; representative images are shown on the right, bar = 200 μm.

Cell migration and invasion are highly dynamic processes that rely on an active actin reorganization. Therefore, the actin cytoskeletal organization was investigated by phalloidin staining, followed by quantification of F-actin filaments/cell or the mean F-actin length. We found no significant differences in the number of actin filaments/cell and in the mean F-actin length in U-87 MG cells in the presence or absence of Mannitou antibody (Figure 4A). However, U-138 MG cells exhibited significantly longer F-actin filaments of 119% ± 2.8% when they were treated with Mannitou antibody compared to the control cells set to 100% ± 2.8% (Figure 4A), proving actin rearrangements in Mannitou antibody treated cells.

### Mannitou antibody influences cell adhesion

As another hallmark of cancer and a regulatory module of cell migration and invasion into surrounding tissues, cell adhesion in the presence or absence of Mannitou antibody was investigated. Interestingly, both cell lines showed a significantly reduced adhesion in the presence of Mannitou antibody compared to the corresponding controls. Adhesion was reduced from 100% in the corresponding control medium to 47.9% ± 4.3% (U-87 MG cells) or 59.6% ± 6.0% (U-138 MG cells), respectively. Importantly, H28 control antibody had no significant effect on cell adhesion of neither U-87 MG nor U-138 MG cells (Figure 4B).

### Mannitou antibody enhances colony formation

To further investigate the role of paucimannosidic epitopes in tumorigenesis, anchorage-independent growth of U-87 MG in the presence or absence of Mannitou antibody was analyzed. Cultivation of cells in the presence of Mannitou antibody significantly enhanced colony formation to 220% ± 24% compared to control medium (normalized to 100% ± 17%). The control antibody H28 slightly reduced the colony formation (65% ± 6%) although this was not significant (Figure 4C).

### Paucimannose is highly present in grade IV glioblastoma multiforme

**Figure 5:**
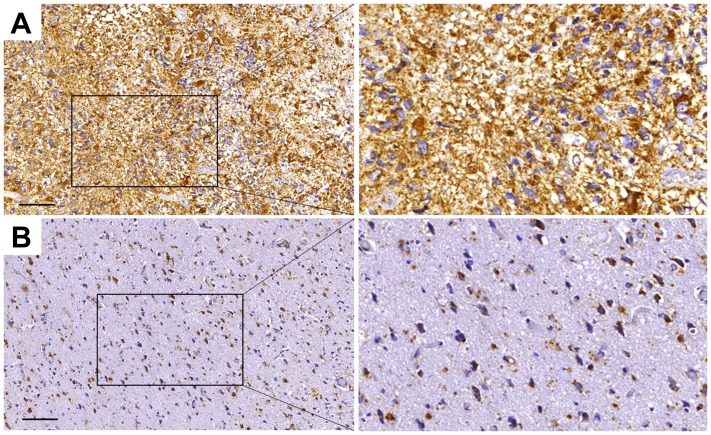
Paucimannose is strongly upregulated in grade IV glioblastoma in humans. Immunohistochemical staining of paucimannose in tumor tissue (**A**) and surrounding, tumor-infiltrated tissue (**B**)**.** Brown areas represent paucimannose immunoreactivity, while blue hematoxylin staining corresponds to cell nuclei, bar = 100 μm.

To provide further support for the involvement of paucimannosidic epitopes in human GBM, an immunohistochemical staining of grade IV glioblastoma and surrounding tissue were performed. Paucimannosidic epitopes was highly present in glioma tissue, with staining of cell somata and processes (Figure 5A). In contrast, the surrounding tissue showed dramatically less staining intensity. The only intensely stained cells in this region were infiltrated tumor cells, reactive astrocytes and activated microglia cells (Figure 5B).

### Neuroblast differentiation associated protein AHNAK carries paucimannosidic epitopes

**Figure 6:**
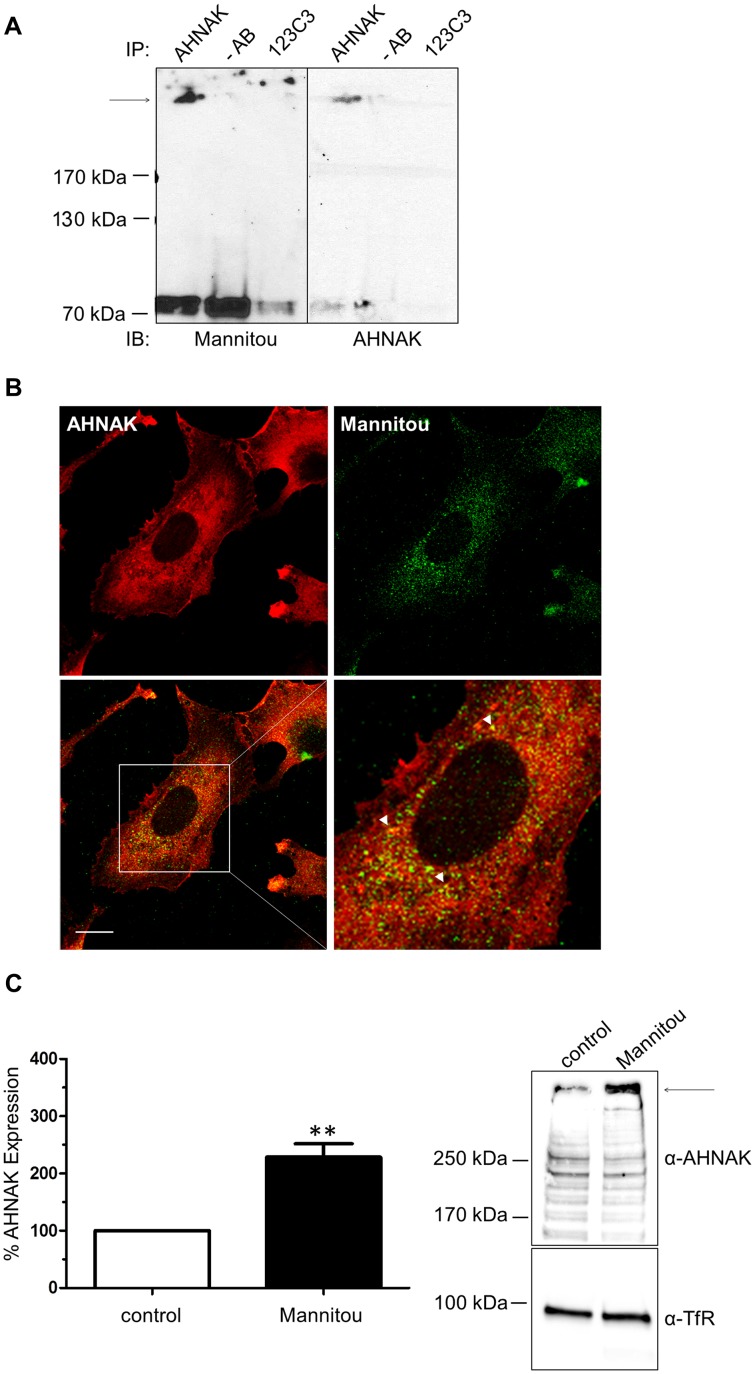
AHNAK carries paucimannose and is upregulated by Mannitou antibody. (**A**) U-138 cell lysates were subjected to immunoprecipitation using AHNAK-specific antibody or 123C3 (recognizing human NCAM) antibody or no antibody (-AB) as controls. Immunoblot analysis was carried out using Mannitou antibody (left). After antibody removal the blot was re-probed with AHNAK-specific antibody (right). The arrow indicates the expected molecular weight of AHNAK. (**B**) U-138 MG cells were fixed and processed for immunofluorescence analysis by visualization of AHNAK with AHNAK-specific antibody and secondary Cy3-conjugated antibodies (red). Paucimannose was visualized using Mannitou antibody and Alexa Fluor 488-conjugated secondary antibodies (green) and images were taken using an LSM510 MetaUV confocal microscope. A partial co-localization can be observed, exemplary highlighted by the arrowheads, bar = 20 μm. (**C**) U-138 MG cells cultured for three days in the presence of Mannitou antibody or control medium. Cell lysates were prepared and equal protein amounts applied onto the SDS gel followed and immunoblot analysis using AHNAK antibody. Antibodies were then removed from the membrane and probed with anti-transferrin receptor (TfR) antibodies as loading control. Quantification was performed from three independent experiments (left) and data are given as mean ± SEM; a representative blot is shown on the right.

Finally, to identify proteins carrying paucimannosidic *N*-glycans, proteins from U-87 MG and U-138 MG cell lysates were separated on an SDS gel. Paucimannose-positive bands were excised and subjected to mass spectrometry analysis. Interestingly, the neuroblast differentiation associated protein AHNAK, also known as desmoyokin, was identified in the U-138 MG cell line. To confirm the potential paucimannosylation of AHNAK an immunoprecipitation was performed. As shown in Figure 6A after precipitation with an AHNAK-specific antibody, paucimannosylated AHNAK could be detected at the expected molecular weight of approximately 629 kDa. Furthermore, a partial co-localization of AHNAK and paucimannosidic *N*-glycans could be observed in immunofluorescence analysis (Figure 6B).

AHNAK has been implicated in cell migration and invasion [27, 28]. Therefore, to investigate if AHNAK is functionally involved in the observed Mannitou antibody-dependent changes in cell migration and invasion, the expression of AHNAK was investigated in control or Mannitou antibody treated cells, respectively. Interestingly, incubation of U-138 MG cells with Mannitou antibody increased AHNAK expression to 229% ± 23% compared to the control, set to 100% (Figure 6C), indicating a functional involvement of AHNAK in the observed effects.

## DISCUSSION

The results presented here identify paucimannosidic epitopes as important regulators of tumorigenic processes in GBM. However, it has to be kept in mind for the subsequent discussion that many results were obtained by incubation of glioblastoma cells with Mannitou antibody, and that the mode of action of the antibody is not known so far. It may be speculated that the application of Mannitou antibody may simply block paucimannosidic glycoepitopes thereby preventing interaction with still unknown *cis-* or *trans*-ligands, or it may activate signal transduction pathways within the cells downstream of the paucimannose carrying protein (s).

Glycoepitopes get more and more into the focus of tumor biology since several studies have highlighted important roles of protein *N*-glycosylation in regulating tumor proliferation, survival, invasion, metastasis and angiogenesis [10, 12]. Changes in *N*-glycosylation are well documented in cancer [8, 29–31]. Consistently, we provide evidence that the specific *N*-glycan paucimannose is highly abundant in aggressive GBM in humans and negatively regulates cell migration, invasion, proliferation and adhesion processes in two glioblastoma cell lines, representing glioblastoma of different aggressiveness. Many of the observed effects were obvious in both cell lines, indicating an involvement of these glycoproteins in cancer processes regardless of their tumorigenicity. This may be explained by the fact that both cell types display relatively high levels of paucimannosidic epitopes although their levels were higher in the more tumorigenic U-87 MG cell line.

Surprisingly, gelatin degradation was only significantly reduced in U-138 MG cells in the presence of Mannitou antibody and not in U-87 MG cells, whereas the Matrigel assay showed reduced invasion in both cell lines. Therefore, it can be speculated that invasion is altered in U-87 MG cells in response to different ECM proteins contained in Matrigel (e. g., laminin, collagen IV, heparin sulfate proteoglycans). However, a tendency of reduced gelatin degradation could also be observed in U-87 MG cells. The reduced gelatin degradation of U-138 MG cells is in agreement with an increase in F-actin stress fiber lengths in U-138 MG cells that may stiffen the cells and thereby reduce cell invasion [32, 33]. Since gelatin is mainly degraded by matrix metallo-proteinases (MMP)-2 and -9 and both MMPs have been implicated in tumorigenic processes [34], the activity of both was also investigated in an MMP activity assay. MMP-9 did not seem to be active since only faint signals could be detected (data not shown). No significant effect was observed on activity of ECM-degrading MMP-2, although a trend to reduced activity in the presence of Mannitou was observed (data not shown), supporting reduced invasion and reduced ECM degradation.

Interestingly, the capacity to form colonies was significantly increased in Mannitou antibody treated cells relative to control cells. One reason for this phenomenon could be the different condition for the cells in the colony formation assay. During the formerly described assays, i. e. proliferation, invasion and migration, cells were attached to the surface of cell culture dishes and in close proximity to each other enabling activation of various signaling cascades. In contrast, the colony formation investigates cell proliferation and the ability of single cells to form colonies anchorage-independently. Additionally, the incubation time with three weeks was much longer in the colony formation assay than in the other assays, in which cells were treated for a maximum of 96 hours. Under these conditions other proteins might be expressed and Mannitou antibody might act differently.

Aberrant protein glycosylation may act on cancerogenic processes through a variety of modes of action, e. g. dysregulation of cell cycle, cell proliferation and growth or degradation of ECM [30]. Although still undocumented, it seems reasonable to speculate that several proteins present in GBM would be carriers of paucimannosidic epitopes that might be affected in their function by Mannitou antibody application. One of the aims of this study was to identify potential paucimannosidic proteins involved in tumorigenic processes in GBM cells. A promising candidate, AHNAK, could be identified in U-138 MG cells. With 629 kDa molecular weight AHNAK is an uncommon large protein. Several cellular locations of this protein have been reported, such as nucleus, cytoplasm and plasma membrane, and it is involved in diverse cellular functions, including remodeling of actin cytoskeleton and cell migration [23]. Despite its size, AHNAK contains only eight potential *N*-glycosylation sites from which six show a high glycosylation probability (NetNGlyc 1.0, [35]). AHNAK has been implicated in cancer progression although its functional role is controversially discussed. It was demonstrated to act as a tumor suppressor in lung and breast cancer and in melanoma. In contrast, an upregulation and involvement in cancer cell migration and invasion in mesothelioma was reported [36–40]. Consistent with the latter hypothesis, AHNAK deficient keratinocytes exhibited reduced migration, invasion and metastatic properties [28]. Even for glioma the results are contradictory. Zhao et al. demonstrated that overexpression of AHNAK reduces proliferation and invasion and increases apoptosis of cells. Furthermore, patients survived longer when AHNAK was expressed at a high level [27]. In contrast, the Human Protein Atlas correlates a higher survival rate with low AHNAK expression (https://www.proteinatlas.org [41]). The results from our model system, where AHNAK is upregulated, and cell migration, invasion and proliferation are reduced in the presence of Mannitou antibody, supporting AHNAK’s role as a tumor suppressor in GBM, are consistent with the data from Zhao et al. [27]. Interestingly, U-138 MG cells express significantly more AHNAK than U-87 MG cells, further confirming the role as a tumor suppressor in glioblastoma (data not shown). These results fit to mRNA data of U-87 MG and U-138 MG cells from the Human Protein Atlas (https://www.proteinatlas.org [41]). Therefore, it seems probable that in GBM not only the generation of paucimannose is regulated but the expression of the protein backbone carrying paucimannosidic epitopes is also highly regulated. Hence, it can be speculated that the functional effects observed are probably attributable to increased ANHAK expression. However, it remains unknown how Mannitou antibodies are capable of increasing AHNAK expression. Mannitou antibody could bind to AHNAK via paucimannose and may either reduce AHNAK degradation or upregulate its synthesis. Recently, an influence of *N*-glycosylation on mono-ubiquitination of CD133 and its subsequent secretion was demonstrated [42], thus, an interplay between paucimannosidic epitopes and degradation processes seems possible. Otherwise, an increase of synthesis would probably be an indirect effect by signal transduction processes, activated by binding of Mannitou antibody to AHNAK, or other not yet identified protein (s) and may involve regulation at transcriptional and/or translational level since we do not know whether AHNAK mRNA levels are changed by Mannitou application or not. Signal transduction could be activated by crosslinking of paucimannose-carrying proteins at the cell surface. Alternatively, the previously described internalization of Mannitou antibody [21] opens another possibility of Mannitou action inside of cells. This aspect needs further investigation in the future.

Consistent with our *in vitro* results paucimannose was specifically detected in humans in GBM tissue supporting its *in vivo* relevance. The presence of paucimannosylation in reactive astrocytes and microglia in the tumor surrounding tissue is not surprising since protein paucimannosylation has also been implicated in inflammatory processes [22].

The spatio-temporal biosynthesis of paucimannosidic structures is still elusive in cancer cells. More knowledge of these aspects is available in human neutrophils where paucimannosidic proteins were firstly shown to reside in the primary granules and were secondly suggested to be generated from complex glycoproteins via the action of β-hexosaminidases [19, 43].

Short truncated glycans have also been observed in cancer and may result from altered expression of various glyco-enzymes involved in the glycosylation machinery [8]. It remains to be explored if the paucimannose-generating β-hexosaminidases are aberrantly expressed or if their coding genes *HEXA* and/or *HEXB* have a high prevalence for deleterious polymorphisms in GBM, aspects that are to be investigated in future projects.

In conclusion, the data reported here provide the first evidence for the functional involvement of paucimannosidic *N*-glycoproteins as a negative regulator of key cancer-related processes in GBM, intriguing findings that may open up for the development of novel therapeutic strategies against and the discovery of glycoprotein biomarkers for GBM.

## MATERIALS AND METHODS

### Cell culture

Hybridoma cells producing Mannitou monoclonal mouse IgM antibody specifically recognizing paucimannosidic structures, hybridoma cells producing H28 antibody (monoclonal rat IgG anti-mouse NCAM antibody, kindly provided by C. Goridis, Institut de Biologie de l’Ecole normale supérieure, Paris, France) or 123C3 antibody (monoclonal mouse IgG anti human NCAM antibody) were cultured as described earlier [21]. For some applications Mannitou hybridoma cells were cultured in Hybridomed DIF1000 (Biochrom, Berlin, Germany).

U-87 MG cells (kind gift from Prof. Pietsch, University of Bonn, Germany) and U-138 MG cells (Cell Lines Services, Eppelheim, Germany) were cultured in DMEM medium with 10% fetal calf serum (FCS), 100 U/mL penicillin, 100 μg/mL streptomycin. U-87 MG cells received additional 2 mM L-glutamine.

### Analysis of doubling time

U-87 MG and U-138 MG cells were plated on poly-L lysine (pLL, 20 μg/mL, Sigma-Aldrich, Taufkirchen, Germany) coated 24-well plates in different cell densities (10000, 20000 and 30000 cells per well, respectively). Cell density was analyzed 4 h after plating and further every 10 or 14 h up to 120 h after plating. At the respective time points cells were directly placed on ice and fixed with 4% formaldehyde. Cells were then stained with 0.1% crystal violet (Sigma-Aldrich) in 2% ethanol for 10 min, washed and air dried. Cell lysis was performed by incubation with 1% SDS. After transfer of the resulting solution into microtiter plates crystal violet staining was assayed at 550 nm. The doubling time was finally calculated using the formula:

$$doubling\;time\;=\;\frac{duration\;x\;log(2)}{log\left(final\;OD550\;nm\right)\;-log(Initial\;OD550\;nm)}.$$

Cells of different plating density and cell proliferation between two time points with three different chosen time periods (e. g. 0-48 h, 24-72 h and 24-96 h) were analyzed. The mean was taken as final doubling time.

### Immunofluorescence analyses

For all experiments cells were plated onto pLL coated coverslips. For analysis of paucimannose in U-87 MG and U-138 MG cells, they were cultured for three days and fixed with 4% formaldehyde followed by blocking and permeabilization with 5% horse serum in PBS/0.5% Triton X-100 for 20 min at room temperature. Cells were incubated with undiluted Mannitou supernatant for 30 min at room temperature before incubation with anti-mouse IgM Alexa Fluor 488-conjugated secondary antibodies (1:500, Dianova, Hamburg, Germany) for another 30 min.

To analyze proliferation-dependent paucimannosylation cells were cultured for three days in the presence of 10 μM AraC (Sigma-Aldrich), fixed and immunofluorescence analysis using Mannitou antibody was performed as described above.

For further analyses of cell proliferation in the presence of Mannitou antibody the cells were cultured for three days in control culture medium without antibody, culture medium containing H28 as control antibody, or culture medium containing Mannitou antibody as described in [21]. Cells were then fixed and permeabilized as described above and incubated with rabbit IgG anti-Ki67 antibodies (Abcam, Cambridge, UK) in a concentration of 1:400 in blocking solution, followed by incubation with anti-rabbit Cy3-conjugated secondary antibodies (1:500, Dianova) for 30 min.

For analysis of co-localization of paucimannose and neuroblast differentiation associated protein AHNAK, cells were cultured for three days, fixed and permeabilized. AHNAK antibody (1:100, Abcam) was preclustered with anti-mouse Cy3-conjugated secondary antibodies in blocking solution (5% horse serum in PBS) for 1 h at 4°C. Identically, Mannitou antibody was preclustered with μ-chain-specific anti-mouse IgM-Alexa Fluor 488-conjugated secondary antibodies. Subsequently, preclustered AHNAK antibodies were added to the cells and incubated 30 min at room temperature. After intensive washing and another blocking step, preclustered Mannitou antibodies were applied and incubated for another 30 min.

In some experiments cell nuclei were stained with Hoechst 33258 (Sigma-Aldrich). All immunofluorescence labelled cells were embedded in Permafluor (Thermo Fisher Scientific, Darmstadt, Germany).

Paucimannosidic glycoepitopes and Ki67-positive cells were analyzed by taking pictures using a fluorescence microscope (Axioplan2, ApoTome system; Zeiss, Germany) with a Plan Apochromat 20×/0.75 objective. To determine the percentage of paucimannose- or Ki67-positive cells, respectively, cells were counted and related to the total cell number determined by nuclear staining.

The intensity of paucimannose-specific staining was analyzed from images taken with the same exposure time by manually outlining paucimannose-positive cells using Image J software (NIH). Integrated density was determined in the selected region and the background was subtracted.

Co-localization of paucimannose and AHNAK was determined by analyzing pictures taken with a Zeiss LSM510 MetaUV confocal microscope equipped with a Plan Apochromat 63×/1.40 oil immersion objective (Zeiss, Oberkochen, Germany).

### Haptotactic migration and invasion assays

U-87 MG and U-138 MG cells were cultured for three days in the presence of Mannitou antibody or in control medium without antibody. Haptotactic migration was performed in transwell Boyden chambers with 8.0 μm diameter pores (#3422, Costar Corp, Cambridge, USA). Cells were detached using HBSS/Na-EDTA (5 mM) and 10000 cells were resuspended in 100 μL Mannitou hybridoma medium or control medium, both containing 5% FCS, and plated on the top of the transwell insert. The bottom chambers were filled with medium containing 15% FCS with or without Mannitou antibody. Cells were allowed to migrate for 16 h (U-87 MG) or 4 h (U-138 MG). To score the percentage of migrated cells, cells from the top or from the bottom side of the filter were removed and the remaining cells were fixed with 4% formaldehyde and stained for 10 min with Hoechst 33258. Cells from randomly selected fields were counted using a 20 × microscope objective on a Zeiss Axiovert 200 inverted microscope (Carl Zeiss). The percentage of migrated cells was calculated as follows:

$$\%\;migrated\;cells=\frac{mean\;number\;of\;cells\;at\;the\;bottom}{mean\;number\;of\;cells\;at\;the\;top\;+\;mean\;number\;of\;cells\;at\;the\;bottom}\;\;.$$

Each sample was assayed in triplicate and the cells from at least five randomly selected fields were counted.

For analysis of cell invasion cells were prepared as described for the migration assay. Invasion was performed for 24 h in the presence or absence of Mannitou antibody in Corning® BioCoat™ Matrigel® invasion chambers with 8.0 μm inserts (#354480, Costar Corp, Cambridge, USA) according to manufacturer’s instructions. The percentage of invaded cells was calculated as described for the cell migration assay.

### Gelatin degradation assay

U-87 MG and U-138 MG cells were cultured for three days in the presence or absence of Mannitou antibody. The gelatin degradation assay was performed according to manufacturer’s instructions (EMD Millipore, Darmstadt, Germany). Briefly, coverslips were coated with pLL followed by activation of the surface with glutaraldehyde allowing covalent binding of the Cy3-coupled fluorescent gelatin. To quench free aldehydes the surface was incubated with cell culture medium. Subsequently, 17.5 × 10^3^ cells were plated onto the surface in Mannitou antibody containing or control medium without antibody, respectively. After 24 h, cells were fixed with 3.7% formaldehyde and permeabilized with 0.25% Triton X-100 in 5% horse serum. Actin cytoskeleton was visualized using FITC-Phalloidin and cell nuclei were counterstained with DAPI. Cells were embedded in Permafluor and images were taken using a LSM810 confocal microscope and a 40 × objective (Zeiss). Degradation of fluorescent gelatin was calculated using image J software (NIH). A threshold was defined and the area of the cells (determined by Phalloidin-staining) and the degraded area (determined by decline of Cy3-fluorescence) were calculated. At least 15 images were analyzed per experiment and results are given in % degradation.

### Analysis and quantification of actin cytoskeleton

Cells were cultured for three days as described before in the presence of Mannitou antibody or control medium without antibody. Cells were then washed and fixed with 4% formaldehyde for 10 min. Permeabilization was performed using 0.1% Triton X-100 for 5 min. Alexa Fluor 488 Phalloidin (Thermo Fisher Scientific) was added to the cells in a concentration of 5 units/mL in PBS for 20 min. Cells were then washed, embedded and confocal images were taken with a Plan Apochromat 63×/1.40 oil immersion objective (Zeiss). The number of actin filaments per cell as well as the length of actin filaments was quantified using FilaQuant-Software (kindly provided by Prof. Konrad Engel and Dr. Harald Birkholz, University of Rostock). Specific adjustments in the program were made as follows: “Top-Hat Radius 7.5, Max. Vertex Laplacian -10”.

### Cell adhesion assay

Cells that were cultured for three days with control medium without antibody, H28 control antibody containing medium, or Mannitou antibody containing medium, were carefully scraped off the culture dish and centrifuged. 2 × 10^4^ cells were plated for 10, 20 or 30 min onto pLL coated 24-well plates. After the indicated time periods media and unattached cells were aspirated, wells were washed once with PBS and remaining attached cells were fixed with 4% formaldehyde for 15 min. Cell attachment was measured quantitatively by crystal violet staining as described above.

### Colony formation assay

For analysis of colony formation of U-87 MG cells a volume of 1.5 mL of 0.5% agar supplemented with control medium, H28 containing medium as control or Mannitou antibody containing medium was plated in 6-well culture dishes. 5 × 10^3^ U-87 MG cells per 6-well dish were mixed with 1.5 mL 0.34% agar supplemented also with Mannitou or H28 supernatant or control medium. A volume of 200 μL of Mannitou antibody, H28 antibody containing medium or control medium without antibody was added on top of the agar and colonies were allowed to grow for 3 weeks in the incubator. Living cells were then stained with nitroblue tetrazolium chloride (Sigma Aldrich) and images were taken with an inverted light microscope.

### Glycomics profiling of the total cell lysates and the microsomal fractions

U-87 MG and U-138 MG cells were cultured as described above. Two cellular fractions were generated for glycomics profiling, i. e. a total cell lysate containing predominantly soluble glycoproteins from the luminal compartments and a matching microsomal fraction containing mostly intracellular membrane glycoproteins [44]. For the former, cells were lysed using RIPA lysis buffer (CC Pro, Oberdorla, Germany) followed by a centrifugation step at 20,000 × g at 4°C for 30 min. The clear supernatants were subjected to acetone precipitation.

The microsomal fractions were prepared largely as previously described [44]. Briefly, cells were harvested in 25 mM Tris-HCl, pH 7.4, 150 mM NaCl, 1 mM EDTA containing leupeptin, pepstatin A, aprotinin and phenylmethanesulfonylfluoride followed by ultra-sonication and centrifugation at 2,000 × g for 20 min. The resulting supernatants were ultra-centrifuged at 120,000 × g for 80 min. The pellets containing the microsomes were washed and fractions were subjected to phase partitioning by incubation at 37°C for 20 min, followed by 1,000 × g centrifugation for 10 min. The upper aqueous layer was carefully removed and the concentration of proteins in the lower detergent phase was determined followed by acetone precipitation. Pellets were dissolved in 8 M urea. Cell lysates and microsomal fractions were generated in three separate preparations for both U-87 MG and U-138 MG cells. The release and preparation of *N*-glycans from the collected fractions, liquid chromatography mass spectrometry/mass spectrometry (LC-MS/MS)-based *N*-glycomics were performed exactly as previously described ([21, 29, 45], see also Supplementary method in Supplementary Materials). *N*-glycans identified in the cell lysates were manually characterized using PGC-LC-MS/MS data based on accurate matches of the theoretical and experimental monoisotopic precursor masses and by the observation of predicted glycan fragment ions in the corresponding collision-dissociation (CID) spectra. The relative and absolute PGC-LC retention times for reduced but otherwise native *N*-glycans were also used for analysis. With this information, the monosaccharide compositions, glycan sequences, topologies and some glycosidic linkages of the observed *N*-glycans could be assigned. Structural features are inferred based on the established knowledge of human *N*-glycosylation biosynthesis pathway and its relatedness to the observed *N*-glycan structures.

### Mass spectrometry to identify paucimannose-positive proteins

SDS-PAGE was performed in duplicate using cell lysates from U-87 MG and U-138 MG cells. One of the two respective gels was subjected to Western Blot analysis using Mannitou antibody, the other was Coomassie stained. Paucimannose-positive bands in the gel were identified by co-localization with signals on the membrane and were excised from the gel followed by peptide preparation for MS analysis. Mass spectrometry analysis was performed by the Core Facility for Mass Spectrometry of the Institute for Biochemistry and Molecular Biology of the University of Bonn. Gel slices were subjected to tryptic in gel digestion [46, 47]. In brief, slices were washed consecutively with water, 50% acetonitrile (ACN), and 100% ACN. Proteins were reduced with 20 mM DTT in 50 mM ammonium bicarbonate and alkylated with 40 mM iodoacetamide (in 50 mM bicarbonate). The slices were washed again and dehydrated with ACN. Dried slices were incubated with 250 ng sequencing grade trypsin at 37°C overnight. The peptide extract was separated and remaining peptides extracted with 50% ACN. Peptides were dried in a vacuum concentrator and stored at -20°C. For subsequent LC-MS analysis peptides were dissolved in 10 μl 0.1% formic acid (solvent A). 2 μl were injected onto a C18 trap column (20 mm length, 100 μm inner diameter) coupled to a C18 analytical column (200 mm length, 75 μm inner diameter), made in house with 3 μm ReproSil-Pur 120 C18-AQ particles (Dr. Maisch, Ammerbuch, Germany). Peptides were separated during a linear gradient from 0% to 35% solvent B (90% ACN, 0.1% formic acid) within 30 min at a flow rate of 300 nL/min. The nanoHPLC was coupled online to an HCT Ultra ion trap mass spectrometer (Bruker Daltonik Bremen, Germany). The five most intense precursor ions were subjected to collision induced dissociation. Fragmented peptide ions were excluded from repeat analysis for 15 sec. Raw data processing was performed with electrospray ionization Compass v1.3 DataAnalysi (Bruker). Database searches with Mascot (Matrix Science Ltd, London, UK) were started from ProteinScape v3.1 against human sequences from SwissProt and cRAP database of contaminants with precursor and fragment ion tolerances of 0.6 Da. Oxidation on methionine and acetylation (N-terminus) were searched as dynamic modifications, propionamide on cysteine as static modification.

### Immunoprecipitation of AHNAK and Western blot analysis

Cell lysates were generated as described above. Supernatants were subjected to immunoprecipitation with anti-ANHAK antibody, 123C3 control antibody or no antibody, and protein G sepharose beads (Sigma Aldrich) overnight at 4°C. Immunoprecipitated AHNAK was eluted by heating in sample buffer (5 min, 95°C) and resulting supernatants were applied onto an SDS gel and subjected to immunoblot analysis using Mannitou antibody and anti-mouse peroxidase (POD)-conjugated secondary antibody (Thermo Fisher Scientific). Positive bands were detected by enhanced chemiluminescence (Western Bright, Biozym, Hessisch-Oldendorf, Germany).

To analyze AHNAK expression in the presence or absence of Mannitou antibody cells were treated with Mannitou antibody or control treated for three days. Cell lysates were prepared as described. Equal protein amounts were applied onto an SDS-PAGE and subjected to Western blot analysis. AHNAK expression was detected with AHNAK-specific and respective secondary POD-conjugated antibodies followed by chemiluminescence. Antibodies were removed from the membrane by incubation with 0.5 M acetic acid/0.5 M NaCl (pH 2.5) for 5 min and re-probed with anti-transferrin receptor antibody (Zymed, San Francisco, USA) as a loading control.

### Immunohistochemical staining for Mannitou

After routine neuropathological diagnostics according the 2016 WHO classifications of tumors of the central nervous system, for immunohistochemical analyses of paucimannose, 4 μm thick sections of the formalin-fixed and paraffin-embedded tumor tissue were used. Immunohistochemical staining was performed with CSA II Biotin-free Tyramide Signal Amplification System (Dako, Carpinteria, USA). To reduce high non-specific background staining, endogenous peroxidases were blocked by incubation in peroxidase blocking buffer for 5 min followed by 60 min protein block at room temperature. Incubation with Mannitou antibody (1:80) was performed overnight at 4°C. Signal amplification and detection was performed using a POD-conjugated secondary antibody recognizing Mannitou antibody and oxidizing a fluorescein-conjugated tyramide (15 min incubation time). This reagent was finally detected by another POD-conjugated anti-fluorescein antibody (15 min) followed by incubation with 3,3′-diaminobenzidine for 1-3 min resulting in a brown, paucimannose-specific staining. Nuclei were stained with hematoxylin (Merck, Darmstadt, Germany).

### Statistical analysis

Statistical analysis was calculated from at least three independent experiments. Two groups were analyzed using the unpaired *t*-test whereas three or more groups were analyzed by one-way ANOVA followed by Tukey post hoc test. Data are presented as the mean ± SEM, or SD for the *N*-glycome profiling. *P*-values < 0.05 were considered statistically significant (^*^ *p* < 0.05, ^**^ *p* < 0.005, ^***^ *p* < 0.001).

## SUPPLEMENTARY MATERIALS



## Abbreviations

ACN: acetonitrile
AraC: β-D-arabinofuranoside
ECM: extracellular matrix
ESI: electrospray ionization
GBM: glioblastoma multiforme
LC: liquid chromatography
MMP: matrix metallo-proteinase
MS: mass spectrometry
pLL: poly-L-lysine
POD: peroxidase
