# Supplementary material for: Paucimannosidic glycoepitopes inhibit tumorigenic processes in glioblastoma multiforme
Source: Oncotarget. 2019 Jul 9;10(43):4449–65. doi: 10.18632/oncotarget.27056 (PMC6633888; doi:10.18632/oncotarget.27056)
Supplement: Supplementary file 1 [file oncotarget-10-4449-s001.pdf]

Paucimannosidic glycoepitopes inhibit tumorigenic processes in glioblastoma multiforme

SUPPLEMENTARY MATERIALS

Supplementary method (Glycomics profiling of the total cell lysates and the microsomal fractions):

For glycomics profiling, proteins were immobilized on a polyvinylidene difluoride membrane (Millipore, Kilsynth, Australia) and the *N*-glycans of bound proteins were released by incubation with 5 U *Flavobacterium meningosepticum* *N*-glycosidase F (Roche, Castle Hill, Australia) for 16 h at 37°C. Released *N*-glycans were incubated with 100 mM ammonium acetate (pH 5) for 1 h at room temperature and dried by vacuum centrifugation. Glycan reduction was performed with 1 M sodium borohydride in 50 mM aqueous potassium hydroxide for 3 h at 50°C, followed by glacial acetic acid quenching. Desalting of *N*-glycans was performed in micro-solid phase extraction formats using strong cation exchange/C18 and carbon columns. Elution was performed with 40% (v/v) acetonitrile containing 0.1% (v/v) trifluoroacetic acid and dried by vacuum centrifugation. *N*-glycans were analyzed by capillary LC-MS/MS (Agilent 1260 Infinity) using an electrospray ionization-ion trap mass spectrometer (LC/MSD Trap XCT Plus Series 1100, Agilent Technologies, Mulgrave, Australia) on a porous graphitized carbon (PGC) capillary column (3 µm Hypercarb KAPPA, 180 µm × 100 mm, Thermo Hypersil, Scoresby, Australia). The separation of the *N*-glycans was carried out over a linear gradient of 0-45% (v/v) acetonitrile/10 mM ammonium bicarbonate for 85 min at a flow rate of 2 µL/min. The sample injection volume was 5 µL. The MS acquisition range was *m/z* 200–2200 in negative ionization mode. The top two most abundant precursors in each spectrum were selected for MS/MS using collision-induced dissociation. Mass accuracy calibration of the mass spectrometer was performed using a well-defined tune mix (Agilent) prior to acquisition. MS and MS/MS data were analyzed and quantified using Compass Data Analysis v4.0 software (Bruker Daltonics, Preston, Australia).

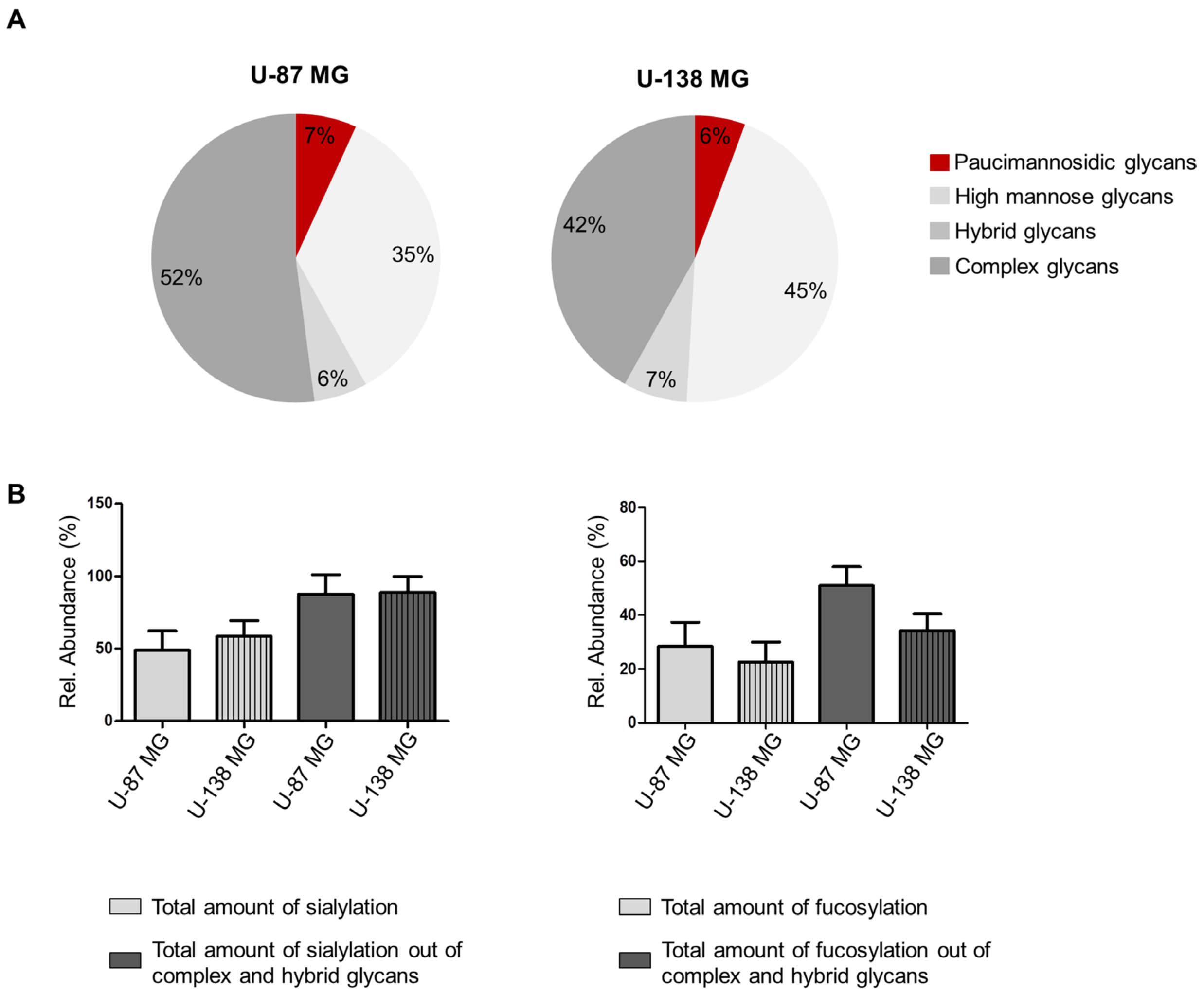

Supplementary Figure 1: (**A**) *N*-glycome profiling of the microsomal fractions of U-87 MG and U-138 MG cells. The relative abundance of the types of *N*-glycans observed in the microsomal fractions is shown. Relative abundance of paucimannosidic *N*-glycans out of the total *N*-glycome is shown in red. (**B**) *N*-glycome profiling of the cell lysates of U-87 MG and U-138 MG cells. The bars represent either the total amount of sialylated (left diagram) and fucoslyated (right diagram) glycans, or their content within the complex and hybrid glycans.
